# Human Melanoma and Glioblastoma Cells Express Cathepsins Supporting Reovirus Moscow Strain Infection

**DOI:** 10.3390/v16121944

**Published:** 2024-12-19

**Authors:** Yulia Ammour, Eugenia Nikolaeva, Olesya Sagimbaeva, Pavel Shamsutdinov, Anastasia Astapenko, Yulia Zhelaeva, Marina Gavrilova, Olga Susova, Aleksey Mitrofanov, Ali Bekyashev, Tatiana Nasedkina, Oxana Svitich, Evgeny Faizuloev, Vitaly Zverev

**Affiliations:** 1 I.I. Mechnikov Research Institute for Vaccines and Sera, 105064 Moscow, Russia; jane.niko.biochem@gmail.com (E.N.); olisiya.s@gmail.com (O.S.); 977876@mail.ru (P.S.); ekzis666@mail.ru (A.A.); yushchetinina@gmail.com (Y.Z.); gavrilovamv@gmail.com (M.G.); svitichoa@yandex.ru (O.S.); faizuloev@mail.ru (E.F.); vitalyzverev@outlook.com (V.Z.); 2 Engelhardt Institute of Molecular Biology, Russian Academy of Sciences, 119991 Moscow, Russia; tanased06@rambler.ru; 3 N.N. Blokhin Russian Cancer Research Center of the Ministry of Health of the Russian Federation, 115478 Moscow, Russia; susovaolga@gmail.com (O.S.); mitrofanov-aa@list.ru (A.M.); abekyashev@gmail.com (A.B.); 4 I.M. Sechenov First Moscow State Medical University of the Ministry of Health of the Russian Federation, 119146 Moscow, Russia

**Keywords:** oncolytic viruses, reovirus, cathepsins, glioblastoma, melanoma

## Abstract

This study evaluates the oncolytic potential of the Moscow strain of reovirus against human metastatic melanoma and glioblastoma cells. The Moscow strain effectively infects and replicates within human melanoma cell lines and primary glioblastoma cells, while sparing non-malignant human cells. Infection leads to the selective destruction of neoplastic cells, mediated by functional viral replication. A positive correlation was identified between viral RNA accumulation and tumor cell death, with no replication observed in non-malignant cells. This study highlights the critical roles of cathepsins B, L, and S as mediators of the oncolytic process. The pharmacological inhibition of these enzymes significantly attenuated reovirus-induced cytotoxicity in melanoma and glioblastoma cells. Conversely, PKR production analysis revealed minimal activation in reovirus-infected tumor cells, suggesting that the hyperactivation of the RAS-signaling pathway and subsequent PKR inhibition do not directly contribute to the selective efficacy of reovirus. Moreover, infected tumor cells exhibited features of both apoptotic and non-apoptotic death, emphasizing the intricate mechanisms of reovirus-mediated oncolysis. These findings underscore the therapeutic promise of the Moscow strain of reovirus as a selective and potent oncolytic agent for targeting melanoma and glioblastoma cells.

## 1. Introduction

Malignant neoplasms represent a significant and escalating challenge to modern healthcare systems. In Russia alone, cancer accounts for nearly 300,000 deaths annually, with approximately 600,000 new cases diagnosed each year [1]. Among the most aggressive and metastatic cancers, human melanoma and glioblastoma exhibit rising global incidence rates, outpacing the growth of other cancer types and imposing a significant socioeconomic burden. While early-stage melanoma can often be effectively treated through surgical intervention, recurrence remains a pressing concern. Notably, approximately 10% of cases present at an advanced, often inoperable or metastatic stage. The five-year survival rate for patients with metastatic melanoma has improved substantially, increasing from less than 10% to 40–50%, owing to advancements in immunotherapy and targeted therapies [2]. However, these malignancies remain highly resistant to conventional treatments, including cytostatic chemotherapy, radiation, and hormonal therapy.

Glioblastomas, characterized by poor prognosis and near-inevitable recurrence, continue to represent a critical therapeutic challenge. Standard treatment protocols encompassing surgical resection, temozolomide-based chemotherapy, and radiotherapy yield a median overall survival of only 14 to 18 months [3]. The high degree of heterogeneity inherent to glioma and glioblastoma cells further complicates treatment strategies, contributing to the frequent failure of immunotherapy and targeted chemotherapies [4,5]. These limitations underscore the urgent need for innovative therapeutic strategies that offer improved efficacy while minimizing toxicity.

Oncolytic virotherapy has emerged as a promising approach in this context, offering the potential to selectively target tumor cells while sparing healthy tissues. This strategy not only directly destroys tumor cells but also enhances anti-tumor immune responses by activating natural killer and cytotoxic T cells, thereby restoring immune surveillance [6].

The concept of oncolytic virotherapy for glioblastoma treatment was pioneered by R.L. Martuza and colleagues, who, in 1991, used herpes simplex virus-1 with attenuated neurovirulence to treat glioblastoma in vivo [7]. To date, herpes viruses, adenovirus, Newcastle disease virus, myxoma virus, measles virus, poliovirus, parvovirus, and Zika virus have the greatest proven oncolytic efficacy [8]. Advances in genetic engineering have enabled the development of viruses with enhanced tropism for brain tissue, capable of crossing the blood–brain barrier and disrupting critical tumor cell processes, such as metabolism, proliferation, and differentiation. Despite these advancements, identifying the most effective oncolytic virus remains challenging due to the molecular and genetic heterogeneity of glioblastoma.

Among oncolytic viruses, the mammalian orthoreovirus has garnered attention for its natural tropism toward specific malignancies, including melanoma and glioblastoma [9]. Notably, reovirus is non-pathogenic to humans, obviating the need for attenuation and making it an attractive therapeutic candidate.

The oncolytic efficacy of reovirus is intrinsically linked to specific mutations within tumor cells, which influence their growth, survival, and immune evasion [10,11,12]. These mutations predominantly involve the hyperactivation of signaling pathways that facilitate viral replication in tumor cells and the overproduction of proteases crucial for viral disassembly within the cell. These mechanisms collectively enable the efficient dissemination of reovirus within tumor tissues, reinforcing its potential as a targeted oncolytic agent.

In early studies exploring tumor cells’ susceptibility to reovirus, the transfection of murine cells with epidermal growth factor receptor (EGFR) significantly enhanced both reovirus replication and virus-induced cytopathic effects [13]. Initially proposed as a direct receptor for reovirus, EGFR was later found to facilitate viral replication by activating Ras-mediated signaling pathway [14]. EGFR hyperactivation is a hallmark of numerous human tumors [15], where it drives Ras signaling to promote tumor proliferation and survival [16]. In addition, Ras expression has been shown to enhance the sensitivity of mouse fibroblasts to reovirus, further implicating this pathway in the selective vulnerability of tumor cells [17].

Clinical trials have demonstrated the safety and therapeutic efficacy of the T3 Dearing (T3D) strain of reovirus, known as Pelareorep or Reolysin, in patients with melanoma (NCT00984464) and glioblastoma (NCT00528684). Approved by the FDA for the treatment of solid tumors and hematologic malignancies, this strain exerts direct cytotoxic effects on cancer cells, while simultaneously activating innate anti-tumor immunity [18,19]. Additionally, reovirus infection sensitizes tumor cells to conventional chemotherapeutic agents and radiotherapy, underscoring it as a promising candidate for combination approach treatments [10]. Ongoing research efforts aim to enhance the efficacy of reovirus-based combination therapies and to evaluate its synergy with immunotherapeutic approaches. Current clinical studies are exploring the integration of reovirus with diverse therapeutic agents to address various human malignancies.

The present study aimed to assess the oncolytic potential of the Moscow strain of reovirus against human metastatic melanoma cell lines and primary glioblastoma cells. This work examines the responsiveness of tumor cells to reovirus-mediated oncolysis, evaluates the kinetics of viral accumulation and tumor cell death following infection, and elucidates the functional mechanisms underlying the selective oncolytic activity of the Moscow strain. Particular focus is placed on identifying cellular factors that modulate sensitivity or resistance to viral oncolysis.

Initially, the Moscow strain was isolated from a component of the medium used for rotavirus cultivation in MA-104 cells [20]. After 5–9 passages in the cell line, the rotavirus RNA disappeared as measured by qPCR [21], while the cells continued to undergo cytopathic changes compared to the control. The SDS-PAGE analysis of the isolated genome RNA revealed the presence of 10 segments in the proportion of 3:3:4 corresponding to the reovirus genome. The consequent sequencing confirmed the reovirus [20]. Thus, as the Moscow strain of reovirus was isolated in trypsin-containing medium, the virus passaging in the absence of trypsin lead to aborted cytopathic effects, indicating the strong dependence of virus reproduction on the presence of trypsin in the nutrient medium. Thus, the characteristics of the Moscow strain according to its dependence on the proteases were of particular interest in this work.

## 2. Materials and Methods

### 2.1. Virus and Cells

The mammalian orthoreovirus Moscow strain (GenBank, N° MH170361) isolated at the I.I. Mechnikov Research Institute for Vaccines and Sera was used in this work [20]. Reovirus with an average titer of 7.48 log_10_ PFU/mL was cultured and titrated in MA-104 cells, and then serially diluted, aliquoted, and stored at −70 °C. MA-104 cells and human lung carcinoma cells, A549, were obtained from the collection of the I.I. Mechnikov Research Institute for Vaccines and Sera.

Differentiated human metastatic melanoma cell lines Mel Ibr (>100 passages) and Mel Z (31 passages) and poorly differentiated cell lines Mel Il (>100 passages) and Mel Mtp (39 passages) (Supplementary Table S1), as well as immortalized human hTERT-BJ fibroblasts and normal kidney epithelial (NKE) cells, were obtained from the collection of the N.N. Blokhin Research Center (Moscow, Russia).

Glioblastoma multiform (GBM) cells Gbl13n, Gbl16n, Gbl17n, Gbl24n, Gbl25n, and Gbl27n at early passages (6–10 passages) were established from primary tumor cells at the N.N. Blokhin Russian Cancer Research Center from the surgical resection or biopsies of confirmed GBM cases and genetically characterized. All patients gave their informed consent. The molecular–genetic profile of cell lines was performed using NGS sequencing. The mutations found are summarized in [22,23].

Melanoma and glioblastoma cells were cultured as described in previous works [21,23].

### 2.2. Infection of Cell Lines with Reovirus

When a cell monolayer reached 80–90% confluency in T25 flasks (Corning, Oneonta, NY, USA), it was rinsed with 3 mL of Hanks’ Balanced Salt Solution (HBSS, Servicebio Technology Co., Wuhan, Hubei, China) solution. Considering the cell count per vial and the initial viral titer, a range of virus dilutions was prepared in DMEM/F12 (Servicebio) supplemented with HEPES (PanEco, Gorki Leninskie, Russia), 1% (*v*/*v*) GlutaMAX (Life Technologies, Paisley, UK), and 1% (*v*/*v*) Pen/Strep (Life Technologies). Subsequently, 2 mL of these dilutions was added to each cell line at varying multiplicities of infection (MOIs). The infected cells were then incubated at a controlled temperature of 37 ± 1 °C in an environment with 5% CO_2_ for 2 h. Control groups included uninfected cells, cells treated with reovirus inactivated by heating at 56 °C for 30 min, and reovirus deactivated by UV irradiation (20,000 μJ/cm^2^ for 1 h). Following the viral adsorption phase, the inoculum was discarded, and the cells were washed with HBSS and cultured in growth medium supplemented with 2% fetal bovine serum (FBS; HyClone, South Logan, UT, USA) at 37 ± 1 °C in a 5% CO_2_ atmosphere for 5 days.

Infected cells were collected daily to determine the kinetics of viral accumulation. Flasks were subjected to multiple freeze–thaw cycles (2–3 times) and clarified through low-speed centrifugation at 3000 rpm for 10 min using an LMC-3000 centrifuge (Biosan, Riga, Latvia). Virus titration was performed using 40 L of the viral-containing sample in 96-well plates (Corning, Kennebunk, ME, USA) at 37 ± 1 °C in a 5% CO_2_. Each sample was inoculated with three replicate wells. At 24 hours post infection, 10 μL of a 4.5% Triton (Sigma, Kawasaki, Japan) solution was added to each well. The plates were covered with a foil seal and placed at −70 °C prior to qPCR analysis.

In parallel, the cell monolayer from a duplicate flask was lysed in 300 μL of RLT lysis buffer (Qiagen, Hilden, Germany) per well, at specific time points: 0, 24, 48, 72, and 96 hours post infection (h.p.i.). The cell lysates were then centrifuged at 1000 rpm for 5 min, and the resultant supernatants were preserved at −70 °C until further examination.

### 2.3. Assessment of Cell Survival by MTT Assay

A total of 100 μL of growth medium containing 1.5 × 10^4^ cells was seeded into each well of 96-well plates. Cells were infected with serial dilutions of virus-containing material, as described above, and incubated at 37 °C in a 5% CO_2_ atmosphere for 3–120 h.p.i. Every 24 h, 25 μL of a solution of 3-(4,5-dimethylthiazol-2-yl)-2,5-diphenyltetrazol bromide (MTT, Promega, Madison, WI, USA) was added as described previously [24]. Optical density analysis was performed on an Anthos Zenith 3100 microplate detector (Anthos Labtec Instruments, Salzburg, Austria) at a wavelength of 570/650 nm.

A total of 100 μL of growth medium containing 1.5 × 10^4^ tumor cells was added to the wells of 96-well plates. After 24–48 h, cells were incubated with a solution of cathepsin B and L inhibitor (Cathepsin Inhibitor I, Calbiochem, EMD Millipore Corp., Billerica, MA, USA) or a pan-inhibitor (Protease inhibitor Cocktail, Sigma-Aldrich, Steinheim, Germany) at a concentration of 5 or 10 μg/mL per well for 1 hour at 37 °C in a 5% CO_2_ atmosphere. Then, the cells were infected with serial dilutions of virus-containing material, and the same manipulations described previously were performed.

### 2.4. Quantitative Reverse Transcription and Real-Time PCR-Based (qPCR) Potency Assay

The triton-lysed infection plates were mixed on a Plate Vortexer for 10 s and centrifuged at 1000 rpm for 1 min. Virus replication was assessed by the viral RNA level using the qPCR method. A commercial One-Step RT-PCR kit (OT-2, Syntol, Moscow, Russia) was used to perform the qPCR according to the kit manufacturer’s recommendations. The forward (CAGCGCGCGCTATTACGATTAC) and reverse (CTGAAGTCCCACCATCAATAAC) primers and fluorescence-labeled probe (ROX-TGCTTTCTTATCAGCGCCCAATGTC-BHQ2) for the qPCR were synthesized at Syntol (Russia). qPCR was performed in a final volume of 25 μL containing 10–50 ng of RNA matrix, a mixture of the forward and reverse primers, and the TaqMan probe with a concentration of 10 μmol, a reaction mixture including buffer solution, 0.5 mM of dNTP, 2.5 mM of MgCl_2_, Hot Start Taq polymerase, reverse transcriptase, and 20 units of RNase inhibitor in two replicates. qPCR was performed on a DTprime5 instrument (DNA Technology, Moscow, Russia). The cycling conditions were as follows: stage 1: 45 °C for 10 min; stage 2: 98 °C for 2 min; and stage 3: 40 cycles—98 °C for 10 s, 55 °C for 10 s, and 72 °C for 30 s. Data analysis was performed according to [25].

### 2.5. Total mRNA Isolation and Reverse Transcription Real-Time Quantitative PCR (RT–qPCR)

Total RNA was isolated from cell lysates using a commercial RNeasy Mini Kit (Qiagen) according to the manufacturer’s instructions. Purified RNA was eluted from the RNeasy Mini Spin column membrane twice with 50 μL of RNase-free water, and the RNA concentration was estimated on a NanoDrop 8000 (Thermo Fisher Scientific, Wilmington, DE, USA) instrument. DNA impurity was removed by treatment with DNAase 20 U (Syntol) for 30 min.

To determine the expression level of target genes, 1 μg of total RNA was incubated at 42 °C for 1 h with the following components (Syntol): 1 unit of reverse transcriptase, 5 μM random hexamers or oligo(dT) primer, 5-fold buffer solution (250 mM Tris-HCl (pH 8.3), 250 mM KCl, 20 mM MgCl_2_, 50 mM DTT), 1 mM of each dNTP and 20 units of RNase inhibitor. The enzyme was inactivated by heating the reaction mixture at 70 °C for 10 min.

The amplification was performed using PCR in a total volume of 25 μL containing 2.5xSYBR Green PCR Master Mix (Syntol), 200 nM of forward and reverse primers for each gene analyzed, and 10–50 ng of the obtained cDNA in two replicates on a DT-Prime5 device according to the following program: 95 °C—15 min, 40 cycles at 95 °C—15 s, and 60°C—30 s.

### 2.6. ELISA

Protein Kinase R (SEA520Hu, Cloud-Clone Corp., Katy, TX, USA) and cathepsins B, L, and S (Elabscience, Wuhan, China) were measured according to the manufacturer’s instructions.

### 2.7. Analysis of Apoptosis in Cell Cultures

Cells were infected with serial dilutions of virus-containing material, as outlined in Section 2.2. Following infection, cells were trypsinized, counted using a Goryaev chamber, and washed with HBSS through gentle agitation or pipetting. Prior to analysis, binding buffer was prepared by diluting a concentrated 4× buffer solution with distilled water. The cells were resuspended in 100 μL of the 1× binding buffer at a density of (2–5) × 10^5^ cells/mL. To each 100 μL of cell suspension, 5 μL of Annexin V-FITC (BD Biosciences, San Diego, CA, USA) and 5 μL of propidium iodide (PI; BD Biosciences, USA) were added. The mixture was incubated for 30 min at room temperature, protected from light. After incubation, the sample volume was adjusted to 400 μL using the binding buffer. Cytometric analysis was performed on a Beckman Coulter EPICS XL Flow cytometer with SYSTEM II software 3.6.0 (Beckman Coulter, Miami, FL, USA). The intensity of the apoptosis reaction was assessed by the dual staining of cells with Annexin V-FITC and PI, analyzing 10,000 cell events per sample. The results were expressed as the percentage of apoptotic cells relative to the total cell population.

In parallel, caspase 3 activity was assessed using a colorimetric method according to the manufacturer’s instructions (E-CKA311, Elabscience) 48 h after the inoculation of the virus. The infection scheme was identical to the scheme described above. The colorimetric signal from each sample was measured using a Varioskan Flash microplate reader (Thermo Fisher Scientific).

## 3. Results

### 3.1. Melanoma and Glioblastoma Cells Undergo Reovirus-Induced Oncolysis

Following the scheme applied in prior studies [22,24], we assessed the sensitivity of human tumor cells to reovirus-mediated oncolysis across four melanoma cell lines (mel Ibr, mel Il, mel Z, and mel Mtp) and five primary glioblastoma cell lines (Gbl13n, Gbl16n, Gbl17n, Gbl24n, and Gbl25n). Notable cytopathic effects, including reduced monolayer density and progressive cytoplasmic granulation, were evident as early as 48 hours post infection (h.p.i.) in tumor cells exposed to reovirus (Supplementary Figure S1).

Cell viability following reovirus infection was quantified using the MTT assay at 24, 48, 72, 96, and 120 h.p.i. across a range of virus dilutions (10^−1^ to 10^−7^). Higher MOIs resulted in a marked reduction in cell viability as early as 24 h.p.i. The effective dose (ED_50_) values varied among melanoma cell lines, with Mel Il, Mel Mtp, Mel Ibr, and Mel Z exhibiting ED_50_ values of 0.7, 1.1, 2.7, and 3.1, respectively.

**Figure 1:**
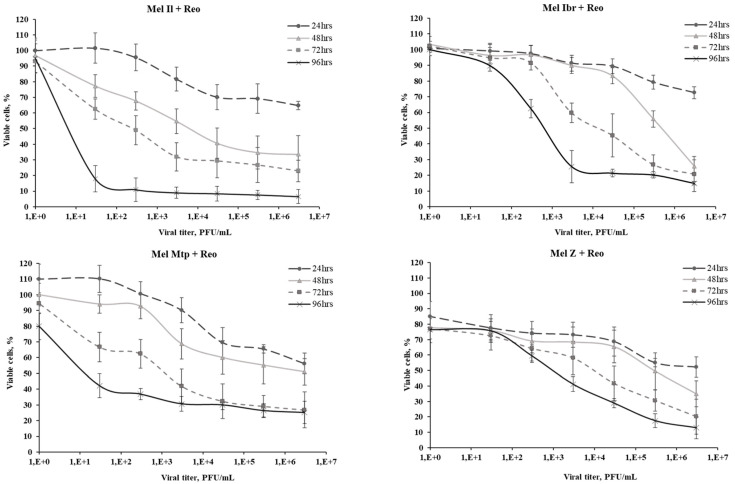

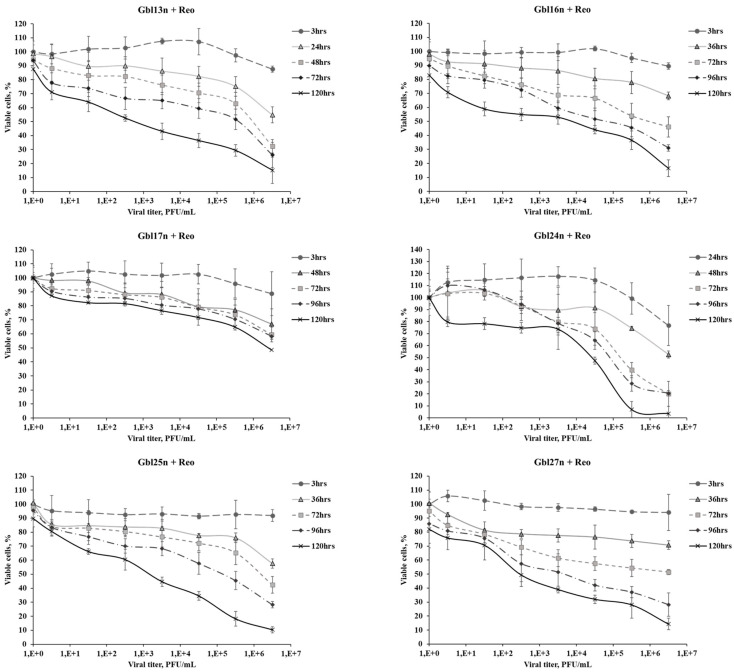
The viability of four melanoma cell cultures and five primary glioblastoma cell cultures incubated with reovirus, Moscow strain, at varying MOIs. The X-axis displays the virus viral titer (PFU/mL) and a control uninfected culture, while the Y-axis indicates cell viability (%). Different colors of the curves represent various time intervals post infection. Cell viability corresponded to the optical density values for the infected cell culture, expressed as a percentage of the values for a similar uninfected culture immediately before infection (0 h). Error bars denote standard deviation.

Primary glioblastoma cells displayed heterogeneity in sensitivity to reovirus-mediated oncolysis, with ED_50_ values ranging from 2.7 to 4.3 log_10_PFU/mL at 120 h.p.i. Among these, Gbl17n cells exhibited the highest resistance, while Gbl24n, Gbl16n, Gbl25n, Gbl13n, and Gbl27n demonstrated progressively increasing sensitivity (Figure 1).

**Figure 2:**
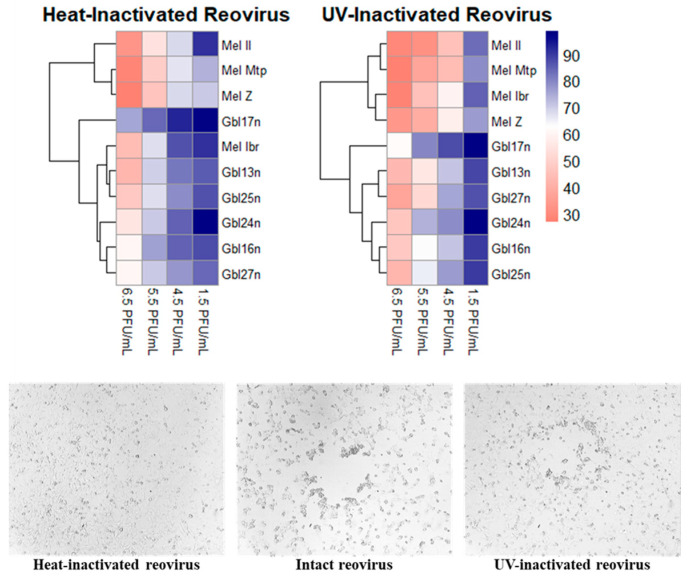
(**Upper panel**) A hierarchically clustered heatmap showing the viability of melanoma cell lines and primary glioblastoma cells 120 h after infection with the Moscow strain of reovirus. Cells were infected at different MOIs (1.5, 4.5, 5.5, and 6.5 PFU/mL). The color scale represents cell viability as a percentage, ranging from 30% (coral) to 100% (navy blue). Dendrograms illustrate the clustering of cell lines and conditions based on viability response. (**Lower panel**) Example of the cytopathic effect of reovirus, Moscow strain, inactivated by UV irradiation or heating on Mel Mtp melanoma cells.

To evaluate the contribution of the reovirus replication to oncolysis, the heat-inactivated and UV-inactivated virus were used, since both lack the ability to replicate within target cells [26]. Cell viability was measured using the MTT assay at 24–120 h post treatment with inactivated virus (Figure 2).

UV inactivation preserved the cytotoxic potential of the reovirus, causing tumor cell death comparable to that induced by a replication-competent virus during early time points. In contrast, heat inactivation significantly reduced cytotoxicity, as evidenced by higher cell viability in treated cells. Thus, UV inactivation preserved the cytotoxic effect of the virus. However, the heat-inactivated virus preserved cell viability at approximately 100%, while the UV-inactivated virus reduced cell viability to 65–70%. However, during further incubation, the replication-competent virus leveled off the difference. By 96 h.p.i., cells treated with replication-competent reovirus showed substantial reductions in viability, 25–40%, whereas heat-inactivated virus treated cells retained 35–75% viability. In contrast, UV-inactivated virus reduced cell viability similarly to the non-inactivated virus, confirming that the UV-treated virus retained a significant degree of oncolytic activity.

**Figure 3:**
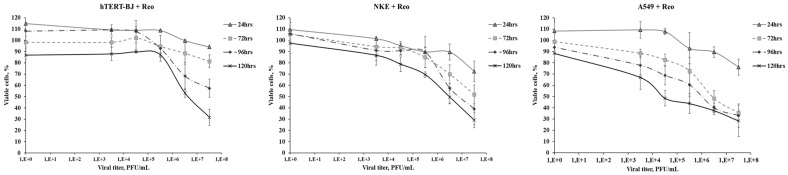
The viability of hTERT-BJ fibroblast cells, human kidney epithelial NKE cells, and A549 lung carcinoma cells incubated with reovirus, Moscow strain, at different MOIs. The X-axis represents time post infection (hours), and the Y-axis shows cell viability (%). The cell viability corresponds to the optical density values for the infected cell culture, expressed as a percentage of the values for a similar uninfected culture immediately before infection (0 h).

To further evaluate the specificity of reovirus-induced lysis, we tested its effects on non-tumor cells, immortalized hTERT-BJ fibroblasts, normal kidney epithelial (NKE) cells, and another malignant cell line, the human lung carcinoma cell line A549. These cell types demonstrated consistent resistance to reovirus infection across a 120 h cultivation period, as shown in Figure 3.

The ED_50_ values for all cell lines used are summarized in Table 1. This observed resistance in certain cell lines may stem from the absence of critical factors required for reovirus-induced oncolysis. Specifically, the inhibition of Protein Kinase R (PKR), a pivotal component of antiviral immunity, might be absent or insufficiently active in these resistant cells [27]. Additionally, these cell types may fail to express the requisite receptors for reovirus on their cellular surface, thereby impeding the virus’s ability to initiate infection [28].

### 3.2. Protein Kinase R Expression Does Not Correlate with Sensitivity to Reovirus-Induced Oncolysis, While EGFR Gene Expression in Glioblastoma Cells Does

Among the melanoma cell lines analyzed, three, Mel Il, Mel Ibr, and Mel Z, harbored hyperactivating mutations in the *BRAF* gene, while one, Mel Mtp, carried a mutation in the *NRAS* gene. In melanoma cells, the activated RAS-signaling pathway is known to inhibit PKR activity through multiple mechanisms, leading in turn to impaired interferon (IFN) responses [29]. Thus, the activated RAS-signaling pathway is considered to be one of the main mechanisms in tumor cells contributing to their sensitivity to reovirus and selective permissiveness [30]. Consequently, PKR may be absent or less abundant in tumor cells relative to reovirus-resistant cells [27].

**Figure 4:**
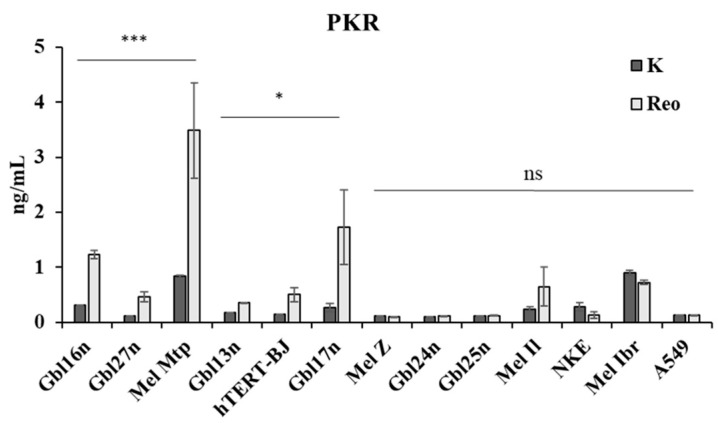
The production levels of phosphorylated PKR in melanoma cell lines, primary glioblastoma cells, and resistant cells: the lung carcinoma cell line, A549, and non-malignant cells, NKE and hTERT-BJ, were assessed analyzed by ELISA. Cell lines were ranged by *p*-values. Each bar represents the mean PKR levels, with error bars indicating the standard deviation (SD). Dark gray bars (K) indicate uninfected cells, whereas light gray bars (Reo) represent reovirus-infected cells. * *p* < 0.05; *** *p* < 0.001; ns—not significant.

To assess the potential correlation, we examined the production of the phosphorylated form of PKR (pPKR) in reovirus-infected cells considered in previous experiments (Figure 4). At an MOI of 1.0, pPKR production was significantly higher in NRAS-mutated Mel Mtp cells, as well as in Gbl16n and Gbl27n (*p* < 0.001) cells. Conversely, reduced pPKR levels were observed in two glioblastoma cells, Gbl13n and Gbl17n, as well as in the normal cell line, hTERT-BJ (*p* < 0.05). RAF-mutated melanoma cell lines, Mel Ibr, Mel Z, and Mel Il; two glioblastoma cells, Gbl24n and Gbl25n; and resistant cell lines, NKE and A549, showed no statistically significant pPKR induction of production following reovirus infection 24 h.p.i. (*p* > 0.05).

These findings indicate that the hyperactivation of the RAS-signaling pathway and the subsequent inhibition of PKR activity do not correlate with reovirus-induced oncolysis or selective tumor cell targeting.

In glioblastoma cells, previous studies have established that the epidermal growth factor receptor (EGFR) is overexpressed in approximately 60% of primary cases, associating with more aggressive tumor phenotypes [31]. EGFR is a crucial player in cellular signaling: its ligand binding triggers receptor dimerization and tyrosine autophosphorylation, ultimately promoting cell proliferation [32,33]. On the other hand, EGFR has been identified as one of the key cell surface proteins recognized by reovirus, specifically binding to the N-terminal extracellular domain of EGFR, as demonstrated by Huang and colleagues [34].

**Figure 5:**
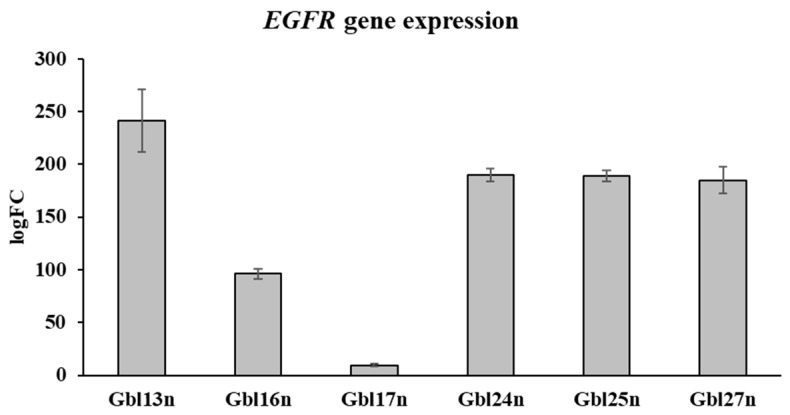
*EGFR* gene (gene ID—ENSG00000146648) expression in glioblastoma cells as measured by mRNA sequencing. Each bar represents mean log fold change, with error bars indicating standard deviation (SD). FC indicates fold change.

To explore the potential role of *EGFR* gene expression in reovirus sensitivity, previous transcriptome analysis was applied to primary glioblastoma cells used in this study. The results, shown in Figure 5, reveal notable heterogeneity in *EGFR* expression among the glioblastoma cell lines. In particular, Gbl13n and Gbl24n displayed a high *EGFR* expression, suggesting increased susceptibility to reovirus-mediated oncolysis, consistent with the receptor’s role in facilitating viral entry. In contrast, the lower *EGFR* expression observed in Gbl17n correlated with the resistance to reovirus oncolysis identified earlier.

### 3.3. Melanoma and Glioblastoma Cells Support the Reovirus Replication

Although *EGFR* gene expression showed potential as a predictor of reovirus susceptibility, the link between susceptibility and permissiveness is not straightforward. While susceptibility refers to the initial entry of the virus into cells, permissiveness involves the ability of tumor cells to support the complete replication cycle of the virus, including viral genome replication, transcription, and the assembly of new virions.

**Figure 6:**
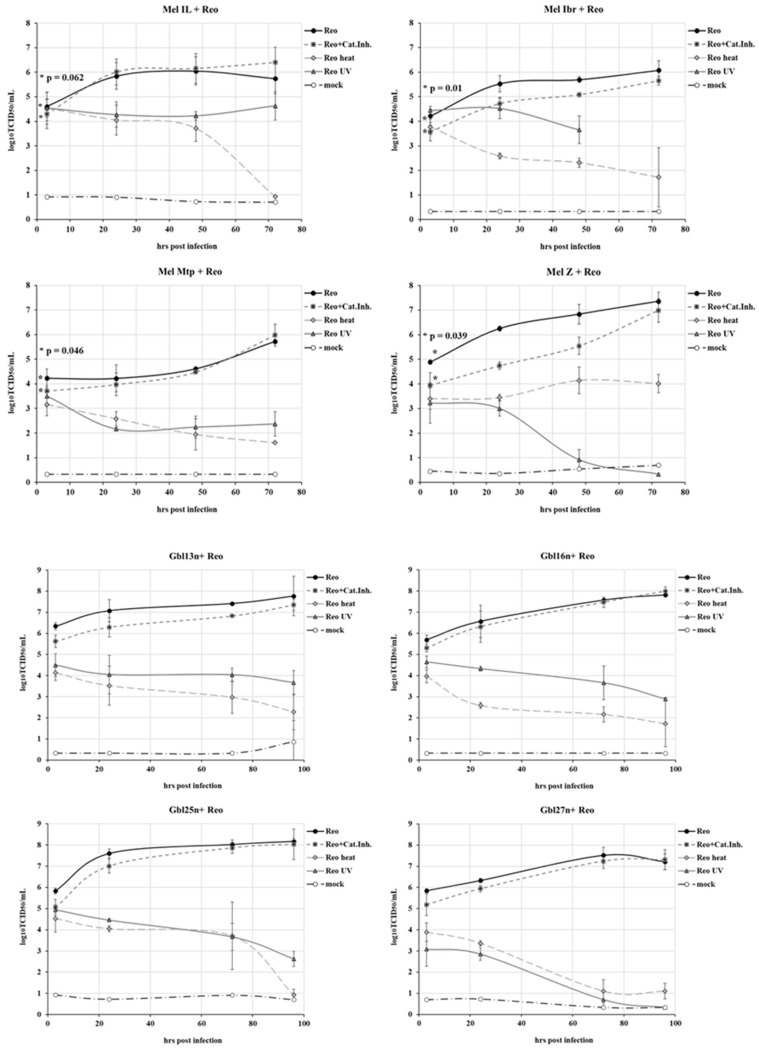

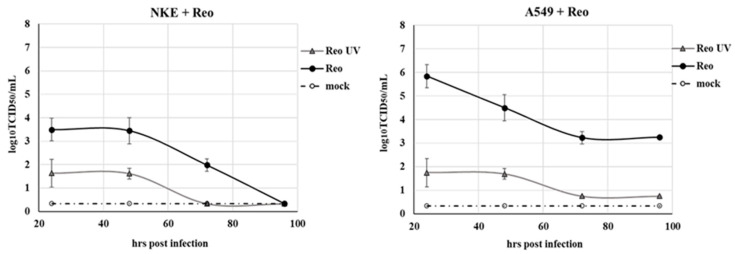
Accumulation of RNA from replication-competent and inactivated (via heating (heat) and UV irradiation (UV)) reovirus intracellularly after infecting melanoma cell lines and primary glioblastoma cells with MOI of 0.1. The X-axis represents the time post inoculation, while the Y-axis indicates the RNA accumulation expressed in log_10_TCID_50_/mL. * indicates *p*-value between RNA accumulation in cells infected with reovirus and infected in the presence of a cathepsin inhibitor.

To determine whether reovirus-mediated oncolysis in glioblastoma and melanoma cells is dependent on viral replication, or if the mere presence of viral RNA alone is sufficient to trigger cell death, we tracked the kinetics of viral RNA accumulation. The permissiveness was assessed using a qPCR-based potency assay allowing us to quantify viral RNA accumulation in infectious virions (Figure 6). Tumor cells were reinfected with reovirus at an MOI of 0.1, and viral RNA accumulation dynamics were monitored over 72 h for melanoma cells and 96 h for glioblastoma cells. In parallel, the replication capacity of functional reovirus was compared to that of heat- or UV-inactivated reovirus. For replication-competent reovirus, qPCR potency assay analysis revealed a time-dependent increase in viral RNA levels, indicating productive infection and, consequently, the permissiveness of tumor cells to reovirus. Conversely, cells infected with heat- or UV-inactivated virus showed no significant RNA accumulation, confirming that heat or UV treatment eliminated the replication capacity.

Thus, the increased cytotoxicity of the UV-inactivated virus was not directly tied to RNA replication. This is likely due to the cross-linking of surface proteins and damage to the viral genome caused by UV exposure, leading to the release of dsRNA during the infection and induction of helicase receptors, which in turn mediate cell death [35].

The A549 cell line further demonstrated resistance to reovirus, as no replication was detected in this culture. However, the replication of reovirus in glioblastoma cells was also found to be minimal, with one cell line, Gbl24n, being unsupportive of the reoviral growth. Thus, these findings indicate that the oncolytic activity of reovirus in glioblastoma cells is not necessarily reliant on active replication.

In summary, all tested melanoma cell lines and four of the five glioblastoma cells demonstrated sensitivity to the Moscow strain of reovirus. In contrast, normal immortalized cell lines, human hTERT-BJ fibroblasts, NKE cells, and two tumor cell lines, A549 and Gbl17n, exhibited relative resistance to reovirus-mediated oncolysis. Furthermore, melanoma cell death strongly depended on the efficiency of viral RNA replication, as evidenced by the correlation between the kinetics of viral RNA accumulation in infected melanoma cell lines and the rate of cell lysis in contrast to cell death mediated by inactivated reovirus. In contrast to melanoma cell lines, cell death in primary glioblastoma cells appeared independent of replication efficiency.

### 3.4. Modulation of Cathepsin Activity as a Factor Mediating Oncolysis of Reovirus

To elucidate the mechanisms driving the oncolytic activity of the Moscow strain of reovirus, we investigated the role of cathepsin activity, a critical factor in the reovirus life cycle. Cathepsins mediate the proteolytic processing of the virus within the tumor cells or their microenvironment, enabling subsequent viral replication [36,37]. Tumor cells, in turn, overexpress cathepsins to promote metastasis, invasion, and angiogenesis [38].

First, we considered the expression of cathepsin genes well known to contribute to glioblastoma development (Table 2).

**Figure 7:**
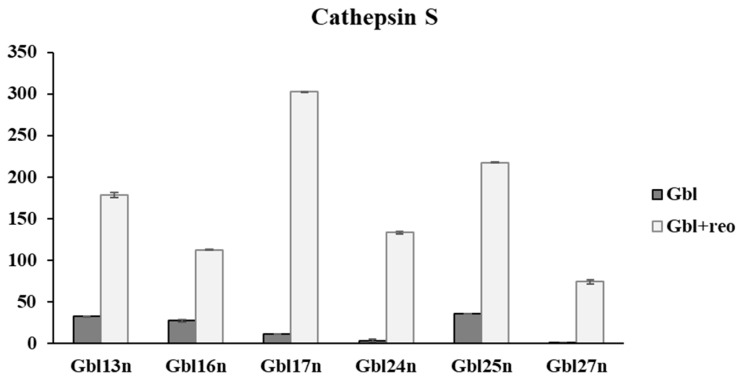
The expression level of the cathepsin S gene mRNA in infected and uninfected glioblastoma cell lines. Cells were infected with the virus at a multiplicity of infection (MOI) of 1.0, and the mRNA levels were assessed at the specified time points using real-time quantitative PCR (qRT-PCR).

The most significantly abundant gene among all was the gene coding cathepsin B. The lowest levels of the cathepsin B gene expression were in cells Gbl17n and Gbl24n, which did not support reoviral replication. However, the statistical analysis revealed the cathepsin S gene as the most differentially expressed between responsive and nonresponsive cells to reovirus-mediated cell killing (*p* = 0.0012). The data were validated by qPCR-RT. Intriguingly, the gene coding cathepsin S was the only gene significantly upregulated among the studied genes after infection with reovirus (p (Spearman) = 4.55 × 10^−5^) in all glioblastoma cell lines (Figure 7). It is known that cathepsin S played an important role in the regulation of autophagy and apoptosis in human glioblastoma cells; on the other hand, this may support acid-independent infection by some reoviruses [39,40].

**Figure 8:**
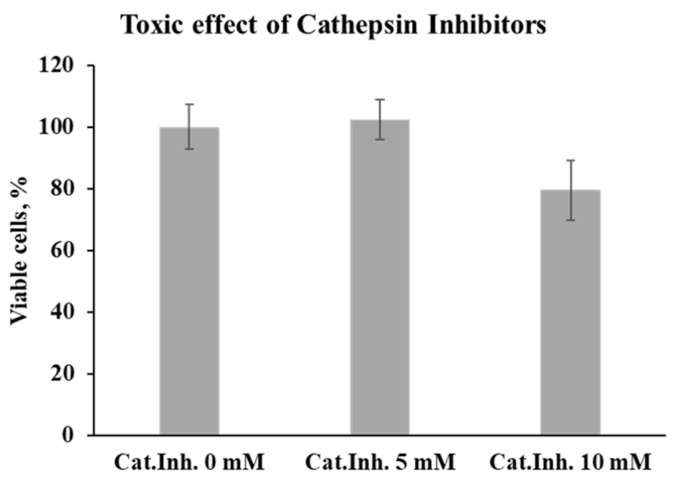
Toxic effect of cathepsin inhibitors on melanoma cells. The toxicity of cathepsin inhibitors was evaluated based on the percentage of viable Mel Il melanoma cells 24 h post incubation with inhibitors of cathepsins B and L.

To evaluate the contribution of cathepsin activity in melanoma and glioblastoma cells, we employed a selective inhibitor targeting cathepsins B, L, and S. First, we assessed the cytotoxicity of the inhibitor on tumor cells by an MTT assay (Figure 8). Working concentrations of 5 mM and 10 mM were selected for further experiments. At 5 mM, the inhibitor showed no toxic effect on tumor cells. However, at 10 mM, a slight reduction in cell viability relative to untreated controls was observed, although this difference was not statistically significant (*p* = 0.069).

**Figure 9:**
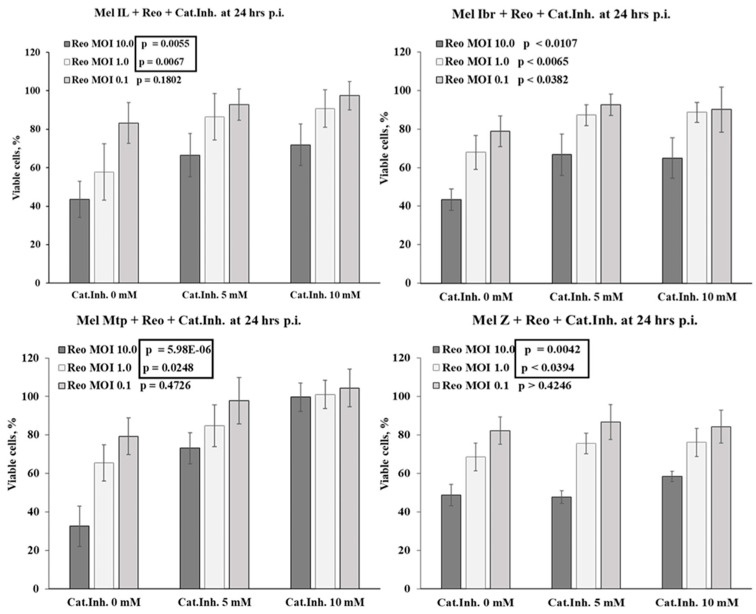

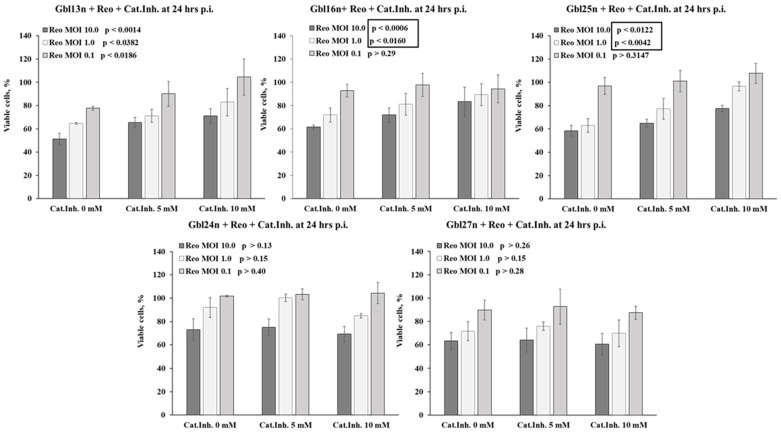
Modulation of cathepsin activity in reovirus-infected melanoma (**upper panel**) and glioblastoma (**lower panel**) cells. The cathepsin-mediated activity was assessed by the percentage of viable melanoma or glioblastoma cells at 24 h post incubation with reovirus at MOIs of 0.1, 1.0, and 10 in the presence of cathepsin inhibitor at concentrations of 5 and 10 mM and without cathepsin inhibitor (0 mM). Black boxes indicate significant differences.

At the next stage, the melanoma and glioblastoma cell lines previously identified as sensitive were infected with reovirus at MOIs of 0.1, 1.0, and 10, in the presence or absence of a selective cathepsin inhibitor. Untreated infected cells served as controls (Figure 9).

At 24 h.p.i., the presence of a cathepsin inhibitor significantly reduced the oncolytic activity of reovirus in all melanoma cells, as evidenced by a marked increase in cell viability compared to the control groups (Figure 9, upper panel). This effect was most pronounced in Mel Mtp cells, where viability in the presence of the 10 mM inhibitor remained equivalent to that of uninfected controls (100%), even at the highest MOI (MOI 10). In Mel Ibr cells, viability increased to 69% and 81% at MOIs of 10 and 1.0, respectively, compared to 43% and 57% in the control groups. A similar pattern was observed in Mel Il cells. However, at 72 h.p.i., these differences had completely leveled out for the higher MOIs, while for the MOI of 0.1, the difference was still significant (*p* = 0.024). The selected cathepsin inhibitor concentrations were sufficient to inhibit the oncolytic activity of reovirus with a MOI of 0.1, whereas at high MOIs, reovirus undergoes partial activation and successfully replicates during the next 48 h. Thus, higher MOIs may overwhelm the viral load that eventually surpasses the inhibitory effect of the cathepsin inhibitor.

For Gbl13n, Gbl16n, and Gbl25n glioblastoma cells, a similar pattern was observed (Figure 9, lower panel). Notably, a significant difference in viability was observed between the infected Gbl13n cells in the presence and absence of cathepsin inhibitor at all MOIs, while in Gbl16n and Gbl25n cells, only at higher MOIs (similar to melanoma cells). However, no significant difference was observed in Gbl24n and Gbl27n cells. Furthermore, Figure 6 illustrates the significant reduction in the reoviral infectivity in Mel Ibr, Mel Mtp, Mel Z, Gbl13n, and Gbl25n cells pretreated with inhibitor, but not in Mel Il, Gbl16n, or Gbl27n cells at 4 h.p.i.

**Figure 10:**
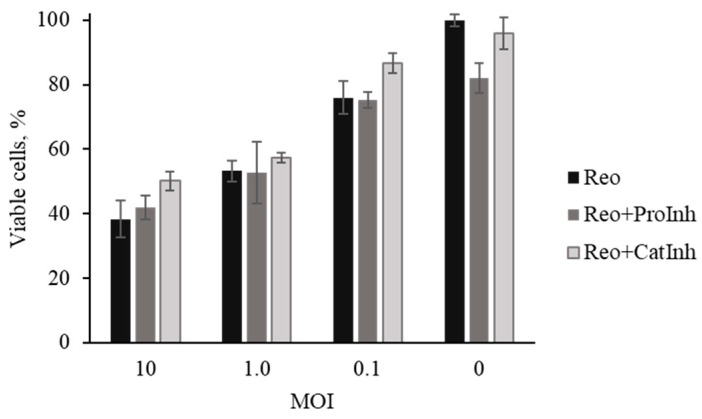
Assessment of the viability of reovirus-infected and uninfected cell cultures following incubation with protease inhibitors. ProInh—a protease inhibitor cocktail; CatInh—an inhibitor of cathepsins B and L; Reo—reovirus.

To confirm the inhibition specificity to the reovirus-mediated oncolysis, a protease inhibitor cocktail including a nonselective cathepsin inhibitor was similarly used. Figure 10 shows Mel Il cell viability after infection with serial 10-fold dilutions of reovirus (MOI 10, 1.0, and 0.1) or in the presence of inhibitors (+Inh): specific (CatInh) or nonspecific (ProInh). The protease inhibitor cocktail had no toxic effect on tumor cells (*p* = 0.07) and no significant differences in viability of tumor cells infected with reovirus at all MOIs (*p* > 0.05).

**Figure 11:**
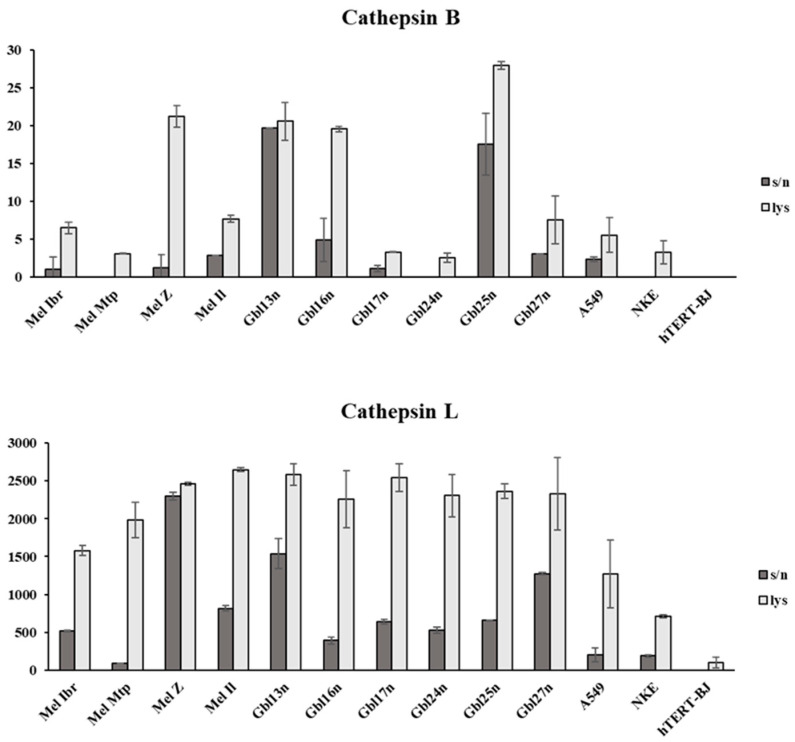

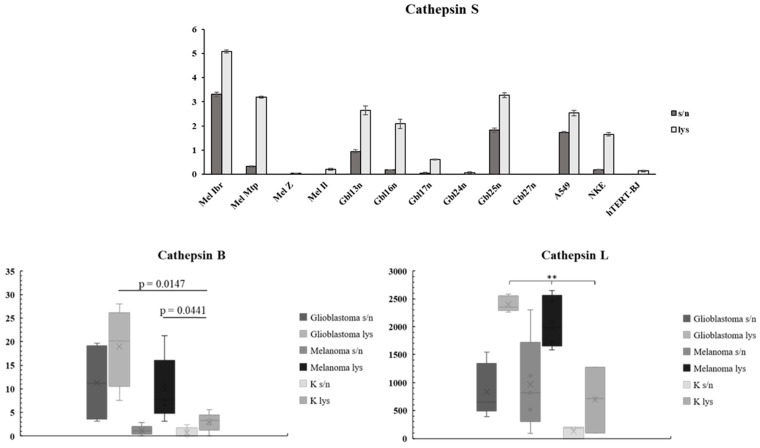
Evaluation of the activity levels of cathepsins B, L, and S in cultures of human melanoma and glioblastoma cells, as well as in resistant cell lines (K): lung carcinoma cells, A549, non-tumorous fibroblasts, hTERT-BJ, and non-tumorous kidney epithelial cells, NKE, in supernatants (s/n) and cell lysates (lys). ** *p* ≤ 0.0057.

The activity levels of cathepsins B, L, and S, key proteases involved in the proteolytic degradation of reovirus’s outer capsid proteins, were assessed by ELISA (Figure 11). Reovirus particles are subjected to proteolytic degradation by cathepsins in the late endosome. However, several studies have reported the membrane-bound and secreted cathepsins B and L, which have not been shown to be involved in the proteolytic degradation of the outer capsid protein [36,37]. Thus, the levels of cathepsins were measured in supernatants as well as in cell lysates.

The levels of activity of the cathepsins intracellularly and in supernatants differed among cell lines. However, reovirus-sensitive cells tended to show higher levels of cathepsins B, L and S activity compared to reovirus-resistant tumor cells (A549) and non-tumor cells (hTERT-BJ and NKE). Thus, the lowest level of cathepsin L activity was observed for the reovirus-resistant lung carcinoma cell culture, A549, as well as non-tumors NKE and hTERT-BJ, both intracellularly and in supernatants. In contrast, reovirus-sensitive tumor cells exhibited significantly higher cathepsin L activity, particularly in the supernatants, with the exception of Mel Mtp cells and other conditionally resistant cell lines. The highest relative level of cathepsin B activity in supernatants was characteristic of Mel Z cells, as well as Gbl13n and Gbl25n, and intracellularly, Gbl13n, Gbl16n, and Gbl25n. Cathepsin B activity was absent in both the lysates and supernatants of hTERT-BJ cells, and was absent in the supernatants of Mel Mtp, Gbl24n, and NKE cells. Furthermore, cathepsin B activity was significantly higher in primary glioblastoma compared with melanoma cells in supernatants, but not in lysates, while for cathepsin L, no differences were observed. Considering the viability data of infected melanoma and glioblastoma (Figure 1) cells, together with control non-tumor cells (Figure 3), it was suggested that both cathepsins contribute to the reovirus infection of tumor cells, in contrast to cathepsin S. Furthermore, for the cells used in this study, the contribution of cathepsin L was more crucial, as cells remained permissive to reovirus-mediated oncolysis in the absence of cathepsin B activity. These results suggest that reoviral proteolytic disassembly by cathepsins is essential for reovirus-induced tumor cell killing. However, no correlation between the level of cathepsin activity and ED_50_ was observed (Table 1). Yet, these results indicate that the activity levels of cathepsins B and L in tumor cells can apparently be used as biomarkers to predict susceptibility to reovirus-induced cell death.

### 3.5. Apoptosis Induction in Melanoma and Glioblastoma Cells in Response to Reovirus Infection

**Figure 12:**
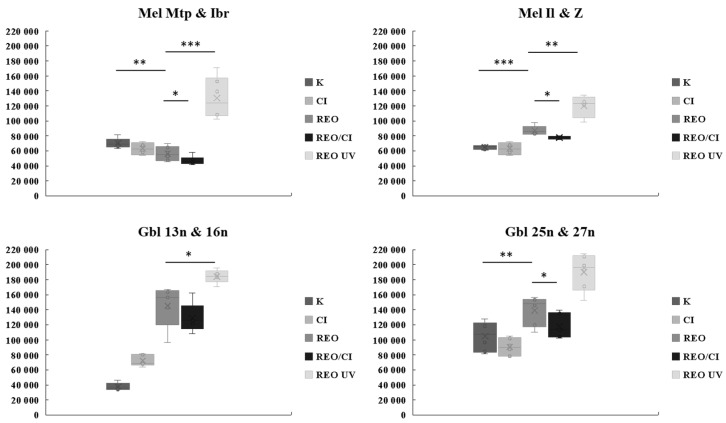
Luminescence analysis of caspases-3 and -7 activity levels in infected melanoma (Mel Mtp, Ibr, Il, and Z) and glioblastoma (Gbl13n, Gbl16n, Gbl25n, and Gbl27n) cells (48 h post infection). * *p* < 0.05; ** *p* < 0.01; *** *p* < 0.001.

To assess cell death, the activity of proapoptotic caspases in infected melanoma and glioblastoma cells was analyzed (Figure 12).

The level of caspase-3/7 activity 48 h.p.i. was significantly increased in glioblastoma cells, pretreated and untreated by a cathepsin inhibitor. No differences were observed between the two groups for Gbl13n and Gbl16n cells, in contrast to Gbl25n and Gbl27n cells. The most significant increase in caspase-3/7 activity was shown for glioblastoma cells incubated with UV-inactivated reovirus. Also, treatment with UV-inactivated reovirus significantly induced caspase-3/7 activation in all melanoma cell lines, indicating that apoptosis signaling was not defective. However, only Mel Il and Mel Z melanoma cells infected with competent reovirus showed a significant increase in caspase-3/7 activity compared to uninfected cells, while in Mel Mtp and Mel Ibr cells, the activity even decreased relative to uninfected cells. Based on these data, cell lines were clustered according to the induction of caspase-3/7 response.

**Figure 13:**
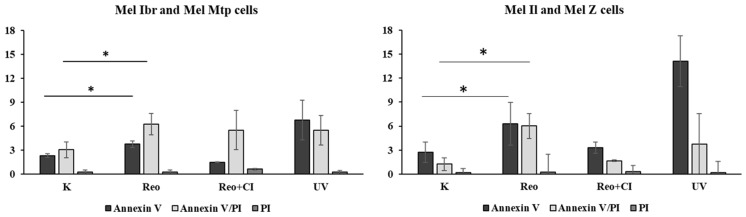

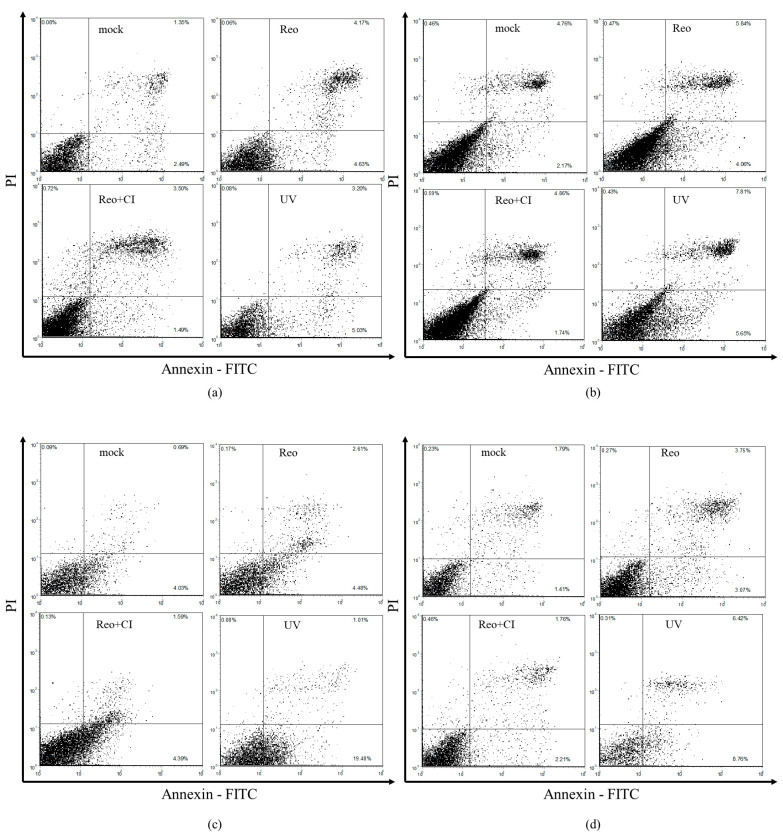
Cytometric analysis of apoptosis in (**a**) Mel Ibr; (**b**) Mel Mtp; (**c**) Mel Z; and (**d**) Mel Il cells. Mock (or K)—uninfected cells; Reo—cells infected with reovirus (24 h.p.i.); Reo+CI—cells pretreated with a cathepsin inhibitor and infected with reovirus (24 h.p.i.); UV—cells incubated with UV-inactivated reovirus (24 h.p.i.). All cells were stained with Annexin V/PI. OX—FITC; OY—PI. * *p* < 0.05.

Next, we performed a cytometric analysis of tumor cells at 24 h after infection in order to capture the early apoptotic events. Uninfected tumor cells, cells incubated with UV-inactivated reovirus, and cells infected with reovirus, pretreated and untreated with a cathepsin inhibitor, were stained with Annexin V and PI (Figure 13).

The proportions of Annexin V+/PI− (suggesting early apoptosis) and Annexin V+/PI+ (suggesting late apoptosis or non-apoptotic death) cell populations were significantly higher compared to uninfected controls. However, in Mel Ibr and Mel Mtp reovirus-infected cells, the population of early apoptotic cells was notably lower than that of the late apoptotic population (Figure 13a,b), whereas no significant difference was observed in Mel Z and Mel Il cells (Figure 13c,d). Overall, the cytometric profiles for cells infected with replication-competent reovirus differed from those incubated with UV-inactivated, particularly in the increased induction of early apoptotic populations in the UV-infected cells. This observation was further supported by the caspase-3/7 activity assay. This difference suggests that replication-competent reovirus infection may disrupt apoptotic signaling, resulting in non-apoptotic cell death.

Thus, it was shown that different tumor cell lines reacted differently to reovirus infection: both apoptotic and non-apoptotic death markers can be characteristic for infected tumor cells. As noted above, neoplastic cells are characterized by specific mutations in genes encoding products of the interferon cascade, leading to the suppression of the interferon-induced inhibition of cell proliferation and, apparently, apoptosis signaling. However, when apoptosis is not possible, necroptosis can be triggered. Furthermore, these results highlight the cellular heterogeneity, although some cell lines can be clustered together.

## 4. Discussion

Mammalian orthoreovirus (reovirus) is a member of the *Spinareoviridae* family, represented by non-enveloped 10-segmented double-stranded RNA viruses. Reovirus infection is widespread; strains serologically identical to human reoviruses have been isolated from a wide variety of animals [20]. Reovirus infection often occurs in humans (with 50–100% of adults showing seropositivity), but in most cases, it is subclinical. Despite its lack of pathogenicity in humans, reovirus displays selective oncolytic activity against malignant cells [41]. The T3 Dearing (T3D) strain of reovirus is currently being tested in clinical trials for several types of cancers [42,43].

In this study, it was shown that the Moscow strain of reovirus effectively replicates in the human melanoma cell lines and primary glioblastoma cells tested, but not in normal human cells or A549 cells. The functional mechanisms of the oncolytic action of reovirus were studied, as well as the main factors of tumor cell sensitivity and resistance to viral oncolysis. Both pre- and post-entry events have been studied in an attempt to define biomarkers that will predict sensitivity or resistance to reoviral oncolysis. In particular, we analyzed PKR production in infected and uninfected cells, as well as *EGFR* gene expression in primary glioblastoma cells and the role of the cathepsins in determining virus mediated cytotoxicity in studied cells. Overall, our data support the results obtained in studies on the T3 Dearing (T3D) strain of reovirus.

Previous studies on the mechanism of reovirus selectivity against malignant and transformed cells showed that Ras signaling pathway activation, including epidermal growth factor receptor (EGFR) and mutated Ras, increased the sensitivity of cells to reovirus-induced cell killing [28]. The activated Ras signaling in these cells was subsequently found to inhibit the function of PKR through multiple mechanisms preventing viral protein translation. Virus-derived double-stranded RNA binds and activates PKR, leading to the phosphorylation of eIF2α. Phosphorylated eIF2α suppresses translation in normal cells. Thus, in Ras-activated cells, dysfunctional PKR signaling allows reovirus replication and subsequent cell death [11]. However, recent studies have demonstrated that reovirus efficiently replicates and kills Ras-activated tumor cells [44]. Thus, these results indicate that there should be a more suitable mechanism as a biomarker for sensitivity to reovirus, compared with the Ras activation status [41].

The overexpression of EGFR and consequent activation of the Ras signaling pathway is the dominant oncogenic process in glioblastoma and melanoma cells, respectively [16,31,32,33]. In normal cells, EGFR can be activated by epidermal growth factor, transforming growth factor α, or other ligands [15]. Upon activation, the dimerization of the EGFR and autophosphorylation of tyrosine residues on the C-terminal domains of the receptor occurs, leading to the activation of proteins downstream in the RAS/RAF/MAPK signaling cascade [45]. EGFR has been shown to be constitutively activated in cells of various epithelial tumors such as in non-small-cell lung cancer, colorectal cancer, and head and neck tumors [46]. In glioblastoma cells, the *EGFR* gene is the most frequently amplified and overexpressed proto-oncogene. EGFR amplification is found in approximately 40–50% of glioblastoma cases [32,33]. Thus, the sensitivity of glioblastoma cells to reovirus could depend on the signaling status in the EGFR/Ras/MAPK pathway.

The data obtained indicate that reovirus predominantly replicated in *p53*- and *PTEN*-mutated glioblastoma cells. We found that Gbl17n cells, the only glioblastoma cells resistant to reovirus-induced oncolysis, lacked *EGFR* gene expression compared to the other primary glioblastoma cells studied. However, we did not observe the correlation of *EGFR* gene expression with the reovirus sensitivity of these cells. Thus, *EGFR* gene expression is mandatory for viral infection, but cannot be considered a biomarker of reovirus selectivity. In this respect, the data are similar to those obtained in other studies [41]. Previous studies reported that two mouse cell lines (NR6 and B82), expressing no EGFR, were relatively resistant to reovirus infection, but the same cell lines transfected with the gene encoding EGFR express significantly higher susceptibility. This enhancement of infection efficiency requires a functional EGFR since it was not observed in cells expressing a mutated EGFR as in tumor cells [47].

On the other hand, reovirus predominantly replicated in NRAS- and BRAF-mutated melanoma cells. An analysis of PKR production revealed no correlation with reovirus-mediated oncolytic efficiencies. Indeed, reovirus efficiently killed the Mel Mtp melanoma cell line that showed significant levels of PKR activation, while A549 and NKE cells that failed to activate PKR were resistant to reovirus infection. Similar results have been reported using other malignant cells [48].

Furthermore, viral replication does not always correlate with cytotoxicity. While the studied cells could support the initial entry of the virus, these cells may lack the ability to support the complete replication cycle of the virus, including viral genome replication, transcription, and the assembly of new virions. Thus, other cellular factors should explain the differences in the cell viabilities following reovirus infection among tumor cell lines.

Virion uncoating is a critical step in the life cycle of reoviruses. After the virus enters the cell, virions undergo proteolysis by cathepsin proteases in endosomes [36], resulting in the formation of infectious subvirion particles [49]. In natural enteric reovirus infections, infectious subvirion particles can also be formed as a result of proteolysis by extracellular proteases, such as gastrointestinal proteases, trypsin, and chymotrypsin [37].

Previous studies demonstrated that cathepsins B and L are highly important for infection with reovirus [50,51]. Furthermore, it was demonstrated that cathepsin S is involved in reovirus infection [40]. Proteases, such as cathepsin family members and matrix metalloproteinases, are often upregulated in tumors and may also be involved in processes other than proteolytic activity during invasion that are associated with tumor progression, such as proliferation, survival, angiogenesis, senescence, apoptosis, and autophagy [52]. In particular, cathepsins A [53], B [54], C [55], D [56], K [57], L [58], S [56], and X/Z [59] are known to be involved in the neoplastic transformation of normal glial cells and associated with immune cells infiltration, immunosuppression, and other processes. Indeed, it was shown that cathepsin B signaling is a driver of glioblastoma malignancy [60]. Furthermore, TGF-beta, which is often overexpressed in a tumor microenvironment, is involved in the induction of the expression of cathepsins B and L, which are important for reovirus infection [61]. In addition, Ras activation is involved in upregulation in the activity of cathepsin family members in tumor cells, suggesting that Ras-activated tumor cells often exhibit high cathepsin activities. Indeed, Y. Terasawa and colleagues demonstrated that Ras-activated tumor cells with low activities of cathepsins B and L are resistant to reovirus, whereas reovirus efficiently exhibited cell killing even though Ras was inactivated only if the cells showed high activity levels of cathepsins B and L [50]. Thus, oncolytic reoviruses exploit specific tumor-associated proteases for proteolytic activation.

As it was mentioned previously, the Moscow strain of reovirus was isolated in MA-104 cells in trypsin-containing medium. The presence of trypsin in the growth medium was a mandatory condition for reovirus reproduction at early passages in MA-104 cells, as the absence of trypsin lead to aborted cytopathic effects [20]. However, long-term reoviral passaging in MA-104 cells allowed us to progressively reduce the trypsin concentration in the growth medium. Therefore, the laboratory-adapted Moscow strain reproduction relies on the presence of trypsin in particular and proteases in general in the growth medium. Q.F. Lin and colleagues demonstrated that the majority of naturally circulating reoviruses rely on intestinal proteases from specific species for uncoating and infection; however, the existence of low-frequency mutations among the natural quasi-species likely enables rapid adaptation to intracellular proteases under selective pressure [62]. Thus, different strains may require different tumor-associated proteases for virus activation. Considering this, the contribution of specific cathepsins to oncolysis mediated by the Moscow strain was also assessed. The reovirus-permissive tumor cell lines (cells susceptible to reovirus oncolysis) exhibited a tendency to express higher levels of cathepsin B and L activities than the reovirus-resistant tumor cell lines. Furthermore, the viability of tumor cells following reovirus infection was not restored by the cathepsin inhibitors to the same degree as the viability of the uninfected tumor cells. In addition to cathepsins B and L as essential factors for the reoviral proteolytic disassembly, the significant upregulation of genes coding cathepsin S in cells sensitive to reovirus-mediated oncolysis was revealed in this work, supporting the results obtained in previous studies [40,50,51]. However, additional studies using siRNA or CRISPR-Cas9 are needed to better define the role of cathepsins in the replication of the Moscow strain of reovirus. Beyond cathepsins B, L, and S, other cellular proteases may also contribute to reovirus-mediated tumor lysis depending on the strain of the reovirus. Naturally circulating reoviruses can vary in their susceptibility to proteolysis by intestinal enzymes from different source species [62]. Furthermore, recently, A. Mohamed and colleagues demonstrated substantial differences in the replication capabilities of highly related T3D laboratory strains in cancer cells in vitro. At the 48-hour post-infection time point, which represents only two rounds of reovirus replication, these differences were already evident, highlighting significant strain-specific variability in replication and tumor cell spread [63,64]. This underscores the importance of directly comparing the T3D and Moscow strains of reovirus in future studies.

In summary, this study demonstrated that the efficiency of reovirus-mediated tumor cell lysis depends on the activity levels of cathepsins, particularly of B, L and S, in tumor cells, suggesting that the activity levels of cathepsins B, L, and S could further be studied as predictive biomarkers for reovirus-mediated oncolysis. These factors may account for the high selectivity of reovirus toward tumor cells with a minimal toxic effect on the surrounding healthy tissues.

## 5. Conclusions

This study demonstrates that the Moscow strain of reovirus effectively replicates in human melanoma cell lines and most primary glioblastoma cells, while exhibiting no replication in non-malignant human cells or the A549 cell line. Notably, all melanoma cell lines tested were sensitive to reovirus infection, whereas sensitivity varied among primary glioblastoma cells. No virus reproduction was observed in non-malignant cells or the A549 cell line. Moreover, reovirus-infected tumor cells exhibited distinct mechanisms of cell death, highlighting the complexity of reovirus-mediated oncolysis.

The functional mechanisms underlying the oncolytic efficacy of the Moscow strain were investigated, identifying key factors contributing to sensitivity and resistance to viral oncolysis. Tumor cell-derived cathepsins B, L, and S were shown to play pivotal roles in facilitating reovirus-mediated oncolysis, as pretreatment with a specific inhibitor significantly delayed the cytotoxic effects of the virus in melanoma and glioblastoma cells.

Thus, these findings underscore the potential of the Moscow strain of reovirus as a selective and effective oncolytic agent, with promising applications in combination therapies targeting metastatic melanoma and glioblastoma.

## Figures and Tables

**Table 1:** ED_50_ for melanoma cell lines, primary glioblastoma cells, A549 and non-malignant cells.

	Mel Il	Mel Mtp	Mel Ibr	Gbl27n	Gbl13n	Gbl25n	Mel Z	Gbl16n	Gbl24n	A549	NKE	Gbl17n	hTERT-BJ
Reovirus	0.67	1.06	2.74	2.74	2.86	3.09	3.14	3.79	4.33	4.83	6.43	6.49	6.84
UV-inactivated	5.20	4.19	4.99	5.88	6.03	6.19	4.19	6.37	6.45	6.85	n/a	6.93	n/a
heat-inactivated	6.48	6.18	6.80	8.42	6.70	7.10	6.18	7.85	7.54	8.00	n/a	9.21	n/a

Colors vary from pale to a bright shade with increasing resistance to reovirus-mediated oncolysis.

**Table 2:** Cathepsin expression levels in uninfected glioblastoma cells.

Name	LogCPM Rank (%)	Gbl13n	Gbl16n	Gbl17n	Gbl24n	Gbl25n	Gbl27n
cathepsin A	93.9	383.5	262.0	327.8	166.9	157.4	117.2
cathepsin B	99.4	1348.7	2146.8	873.2	764.8	1068.7	1454.0
cathepsin C	83.5	206.5	119.8	60.2	55.3	138.8	98.7
cathepsin D	96.8	394.5	410.1	1078.4	152.4	331.1	78.1
cathepsin K	85.8	6.1	28.3	29.0	84.2	8.1	30.1
cathepsin L	94.3	283.0	361.8	129.6	147.1	106.2	64.7
cathepsin S	91.6	33.1	27.5	11.1	3.4	35.3	1.4
cathepsin Z	87.8	96.6	75.8	65.0	107.6	125.1	258.0

CPM—counts per million reads. Numbers represent logFC (fold change) for each cell line. A blue-to-red color heatmap illustrates per-sample gene expression profiles normalized to the average value (geometric mean) across all samples, per each gene.

## Data Availability

Data are contained within the article and Supplementary Materials. Additional information is available on request.

## Supplementary Materials

The following supporting information can be downloaded at https://www.mdpi.com/article/10.3390/v16121944/s1: Table S1: Characteristics of melanoma cells used in the study; Figure S1: The cytopathic effect of reovirus, Moscow strain, on melanoma cells at 48 h post infection.
