# Supplementary material for: Human Melanoma and Glioblastoma Cells Express Cathepsins Supporting Reovirus Moscow Strain Infection
Source: Viruses. 2024 Dec 19;16(12):1944. doi: 10.3390/v16121944 (PMC11680368; doi:10.3390/v16121944)
Supplement: Supplementary file 1 [file viruses-16-01944-s001.zip › Supplementary_Fig._S1.pdf]

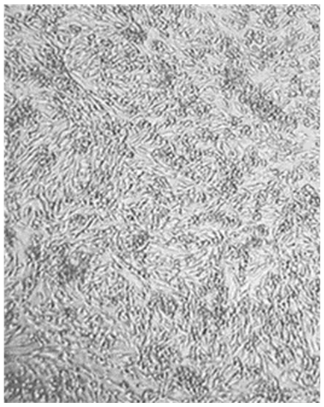

A

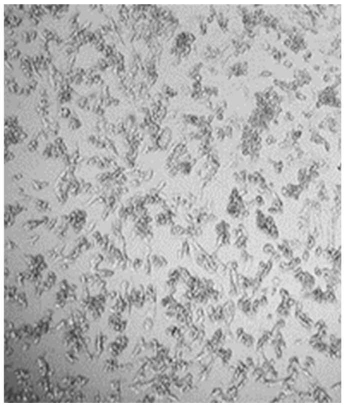

B

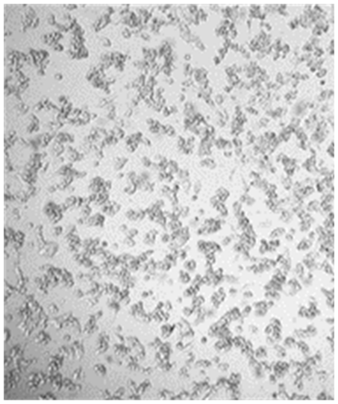

C

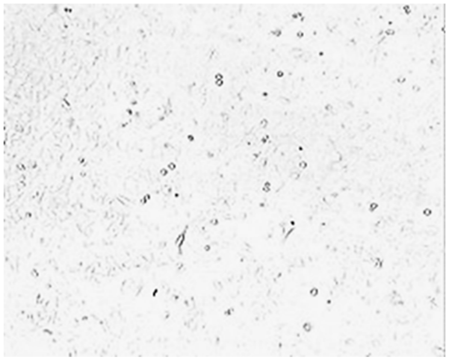

D

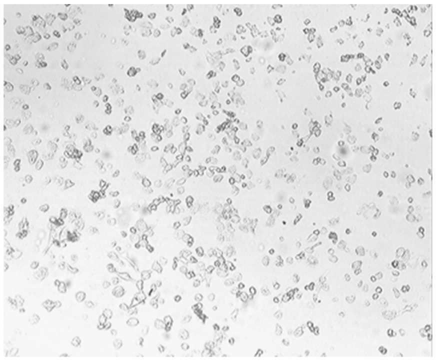

E

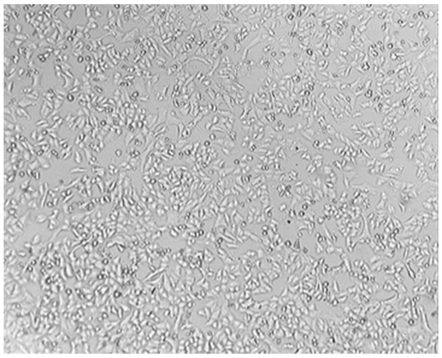

F

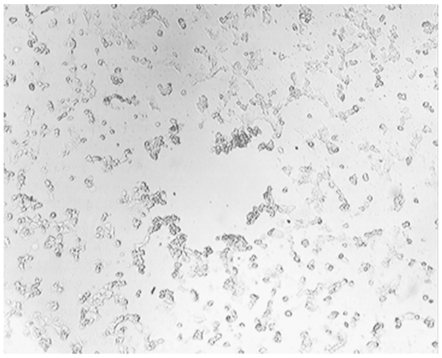

G

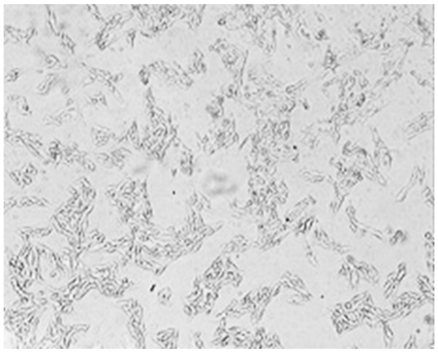

H

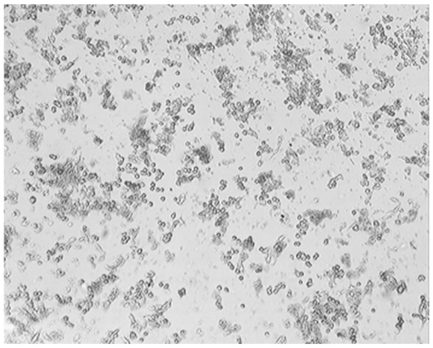

I

Figure S1. The cytopathic effect of reovirus, strain Moscow, on melanoma cells at 48 hours post-infection (Inverted microscopy pictures, 10x magnification). Mel Il cells: (A) Uninfected; (B) infected at a MOI of 0.1; and (C) infected at a MOIs of 1.0 infectious units/cell. **Mel Ibr cells**: (D) uninfected; and (E) infected at a MOI of 1.0. **Mel Mtp cells**: (F) uninfected; and (G) infected at a MOI 1.0. **Mel Z cells**: (H) uninfected; and (I) infected at a MOI of 0.1.
