# Supplementary material for: Human Melanoma and Glioblastoma Cells Express Cathepsins Supporting Reovirus Moscow Strain Infection
Source: Viruses. 2024 Dec 19;16(12):1944. doi: 10.3390/v16121944 (PMC11680368; doi:10.3390/v16121944)
Supplement: Supplementary file 1 [file viruses-16-01944-s001.zip › Supplementry_Table_S1.pdf]

Supplementary Table S1. Characteristics of melanoma cells used in the study

Cell line	Number of passages before use	Differentiation	Mutation	Expression of melanoma and neoplasm markers/TAA
**Mel Il**	**> 100**	Differentiated	BRAF V600K<br>BRAF R389C<br>TP53 F109S	CD63, HMB45, HMW, MelanA, Tyrosinase; MAGE-3
**Mel Ibr**	**> 100**	Poorly differentiated	BRAF V600E	CD63, HMW; MAGE-3
**Mel Mtp**	**39**	Poorly differentiated	NRAS Q61R	CD63, HMW; MAGE-3
**Mel Z**	**31**	Differentiated	BRAF V600E<br>PDGFRA S478P	CD63, HMB45, MelanA, Tyrosinase; MAGE-3
